# Prospective: Evolution of Chinese Medicine to Treat COVID-19 Patients in China

**DOI:** 10.3389/fphar.2020.615287

**Published:** 2021-02-25

**Authors:** Jieya Wu, Baoguo Sun, Li Hou, Fulan Guan, Liyuan Wang, Peikwen Cheng, Sophia Scobell, Yung-Chi Cheng, Wing Lam

**Affiliations:** ^1^ Department of Pharmacology, Yale University School of Medicine, New Haven, CT, United States; ^2^ Department of Oncology and Hematology, Dongzhimen Hospital, Beijing University of Chinese Medicine, Beijing, China; ^3^ Department of Traditional Chinese Medicine, The First Affiliated Hospital of Sun Yat-Sen University, Guangzhou, China; ^4^ Institute of TCM and Health Development, Jiangxi University of Traditional Chinese Medicine, Jiangxi, China; ^5^ Yiviva, Inc., New York, NY, United States; ^6^ Department of Biology, Wesleyan University, Middletown, CT, United States

**Keywords:** COVID-19, Chinese medicine, 3-drugs-3-formulas, pathogenesis, cytokine storm, RAS-mediated bradykinin storm

## Abstract

During the outbreak of the novel coronavirus disease (COVID-19), the Chinese government took a series of public health measures to tackle the outbreak and recommended six traditional Chinese medicine (TCM) evolved formulas, collectively referred to as “3-drugs-3-formulas”, for the treatment. In this prospective article, we will discuss how these six formulas evolved from TCM and what their underlying mechanisms of actions may be by evaluating the historical usage of the component formulas, the potential targeted pathways for the individual herbs used by STAR (signal transduction activity response) database from our laboratory, and the pathogenesis of COVID-19. Five of the six recommended formulas are administered orally, while the sixth is taken as an injection. Five classic categories of herbs in the six formulas including “Qing-Re”, “Qu-Shi”, “Huo-Xue”, “Bu-Yi” and “Xing-Qi” herbs are used based on different stages of disease. All five oral formulas build upon the core formula Maxingshigan Decoction (MD) which has anti-inflammatory and perhaps antiviral actions. While MD can have some desired effects, it may not be sufficient to treat COVID-19 on its own; consequently, complementary classic formulas and/or herbs have been added to potentiate each recommended formula’s anti-inflammatory, and perhaps anti-renin-angiotensin system (RAS)-mediated bradykinin storm (RBS) and antiviral effects to address the unique medical needs for different stages of COVID-19. The key actions of these formulas are likely to control systemic inflammation and/or RBS. The usage of Chinese medicine in the six formulas is consistent with the pathogenesis of COVID-19. Thus, an integrative systems biology approach—combining botanical treatments of conventional antiviral, anti-inflammatory or anti-RBS drugs to treat COVID-19 and its complications – should be explored.

## Introduction

In late December 2019, pneumonia clusters from unknown causes were reported in Wuhan, China. A novel β-coronavirus strain, belonging to the same family as the SARS-associated coronavirus (SARS-CoV) and Middle East Respiratory Syndrome coronavirus (MERS-CoV), was identified as the cause of these pneumonia outbreaks (Petrosillo et al., 2020). This novel β-coronavirus was named 2019 novel coronavirus (2019-nCoV). Its entire viral genome sequence was uploaded to virological.org and GenBank by a consortium led by Yong-Zhen Zhang (Zhang and Holmes, 2020) on January 11, 2020. Subsequently, the International Committee on Taxonomy of Viruses (ICTV) renamed the strain “Severe Acute Respiratory Syndrome Coronavirus 2 (SARS-CoV-2)” on February 11, 2020 and the World Health Organization (WHO) announced “COVID-19” as the official name of this new disease.

At the start of the COVID-19 outbreak, there were no effective treatments available. The Chinese government referenced and applied lessons learned from the SARS outbreak in 2003 and took several bold actions to control COVID-19. First, to slow down and prevent infections, on January 23, 2020, the Chinese government unprecedently locked down a city. Wuhan, with a population of 10 million people, required mask-wearing and enforced a stay-at-home order. This policy was then extended to all high-risk areas across China. Second, local governments constructed new hospitals and added hospital beds to treat severe COVID-19 patients, and remodeled large public facilities into quarantine centers for asymptomatic carriers and mild COVID-19 patients. Third, Chinese scientists isolated and characterized SARS-CoV-2 and then developed and mass-produced detection kits for SARS-CoV-2 to speed up screening. Fourth, researchers from different institutions developed *in vitro* and cell culture methods to test all available current medicines and TCM herbs (based on their historical usages) to identify potential treatment candidates for the range of unmet clinical needs of COVID-19 patients at different stages of disease progression. Researchers also developed new TCM based treatment protocols. Clinicians began examining the potential of herbal formulas to prevent infection and to treat COVID-19. Fifth, scientists began using the viral sequence to explore vaccine approaches. After three months of concerted effort, China was able to significantly reduce COVID-19 deaths. On March 19th, Wuhan reported no new COVID-19 cases.

Currently, the Chinese government recommends six formulas for the treatment of COVID-19, collectively referred to as “3-drugs-3-formulas”. Of these formulas, one is an injectable form and five are administered orally. In this discussion, we will share our perspective on how the six formulas evolved from TCM and we will discuss some scientific basis to support the ways in which these formulas could treat different stages of COVID-19.

## PATHOGENESIS AND CONVENTIONAL TREATMENT OF COVID-19

**FIGURE 1:**
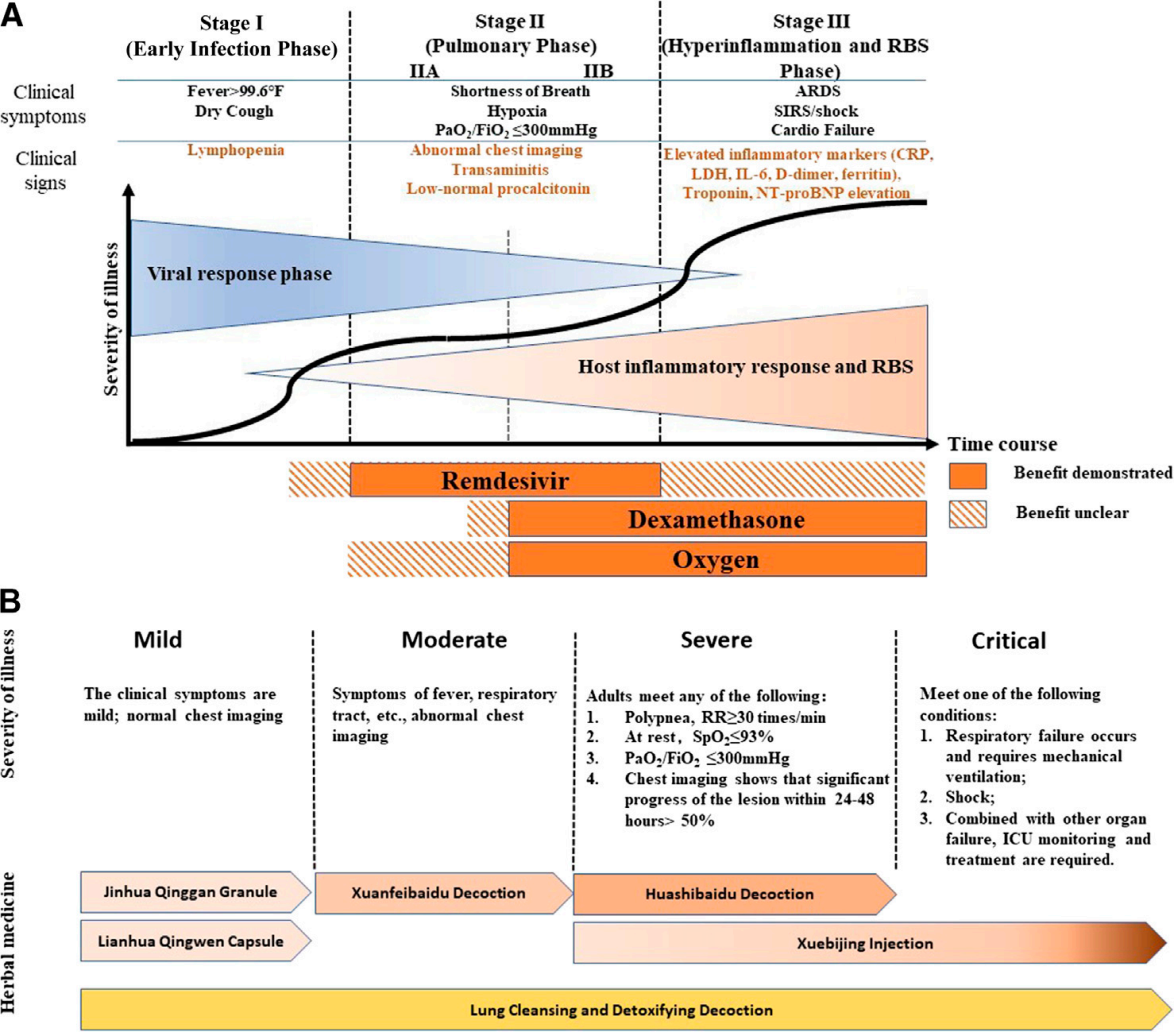
COVID-19 Therapies at Different Stages **(A)** Modified from publication by Siddiqi, H.K., and Mehra, M.R. (2020). COVID-19 illness in native and immunosuppressed states: A clinical-therapeutic staging proposal. J Heart Lung Transplant 39**,** 405–407 **(B)** Modified from China, N.H.C.O.T.P.S.R.O. (2020a). "Diagnosis and Treatment of COVID-19 (8th trial edition)", (ed.) N.H.C.O.T.P.S.R.O. China (Beijing: National Health Commission of the People's Republic of China), and Wang et al., (2020). Comprehensive Analysis of TCM Diagnosis and Treatment Schemes for COVID-19 in All Regions of China. Modernization of Traditional Chinese Medicine and Materia Medica-World Science and Technology 22**,** 257–263. The diagnostic criteria of children in severe stage is deleted in this figure.

COVID-19 patients may exhibit a wide range of symptoms as the disease progresses. Common mild and moderate symptoms include fever, dry cough, fatigue, loss of smell, and diarrhea (Farah Yusuf Mohamud et al., 2020). In severe and critical stages, patients may develop pulmonary symptoms such as acute respiratory distress syndrome (Jin et al., 2020). Patients with pre-existing conditions, such as hypertension, obesity, and diabetes, would exhibit a higher risk of disease progression and lower survival rate (Jordan et al., 2020). Men infected with COVID-19 have lower immune response ability and a higher mortality rate compared to women (Scully et al., 2020). This could also be attributed to sex-determining gene expression, chromosomes, and/or hormones (Li et al., 2020). When COVID-19 progresses to a severe stage, significantly higher plasma levels of inflammatory related cytokines, including IL-1, IL-6, IL-7, G-CSF, IP-10, MCP1, MIP1α and TNFα were found, resulting in cytokine storms (Figure 1A). Studies have suggested that cytokine storms correlate directly with lung injury, multi-organ failure, and an unfavorable prognosis of severe COVID-19 cases (Huang et al., 2020; Ragab et al., 2020; Ruan et al., 2020). Therefore, tackling cytokine storms in the later-stages of COVID-19 might be key to shortening the course of the disease, decreasing mortality rate, and improving the prognosis of COVID-19 patients. Recent research suggests that renin-angiotensin system (RAS)-mediated bradykinin storm (RBS) plays a key role in the pathogenesis of COVID-19 (Garvin et al., 2020). This is now being further investigated.

Physical life support systems such as ventilators, extracorporeal membrane oxygenation (ECMO), artificial liver support systems (ALSS), and blood purification systems, are commonly used to support the life of COVID-19 patients. Drugs used for treatment of COVID-19 in different parts of the world, particularly Western medicine-practicing countries, may vary greatly due to the nature of, and the principles behind the medicines used.

Treatment of COVID-19 in countries practicing Western medicine are based on a pathogenesis perspective. Disease progression can be separated into three stages: early infection, pulmonary phase, and severe hyperinflammation and RBS phase (Figure 1A). The antiviral drug, Remdesivir, demonstrates its benefit when used as a treatment during the pulmonary phase, while the anti-inflammatory drug, dexamethasone, combined with oxygen, has been used to treat the pulmonary to hyperinflammation and RBS stages (IIB amd III) of COVID-19 (Figure 1A) (Siddiqi and Mehra, 2020). There is some dispute over the efficacy of Remdesivir as some clinical trials have failed to show any clinical benefits (Wang et al., 2020c; Spinner et al., 2020). However, Beigel et al. suggested that Remdesivir was superior to placebo in shortening recovery time in adults hospitalized with COVID-19. They also showed evidence of Remdesivir reducing respiratory tract infection (Beigel et al., 2020). Convalescent plasma and monoclonal antibodies may be helpful for severely ill patients as a passive antibody treatment; however, more rigorous clinical trials are needed to prove this (Roback and Guarner, 2020). Recently, a new hypothesis, RBS, has been developed to explain the wide range of symptoms caused by COVID-19 infection. The RBS theory could account for the increased vascular permeability that causes fluid leakage into lung tissue (Garvin et al., 2020). Thus, it has been suggested that existing FDA-approved pharmaceuticals for treating RBS including Vitamin D may be useful for reversing or treating COVID-19. It should be pointed out that angiotensin-converting enzyme 2 (ACE2) is not only a SARS-CoV-2 receptor but it is also part of the RAS-bradykinin axis. Some treatments targeting ACE2 may not only impact viral life cycle but may also play into RBS.

## “3-drugs-3-formulas” for Treating Different Stages of COVID-19

In China, COVID-19 belongs to the “plague” category in TCM and is classified into four stages (mild, moderate, severe, and critical) based on the severity of illness and the symptoms that present (Figure 1B) (China, 2020a). According to China’s guidelines (China, 2020a; Wang et al., 2020), the following six treatments are used for different stages of COVID-19 (Figure 1B): Jinhua Qinggan Granule (JQG) and Lianhua Qingwen Capsule (LQC) are recommended for mild cases, Xuanfeibaidu Decoction (XD) for moderate cases, Huashibaidu Decoction (HD) for severe cases, Xuebijing Injection (XI) for severe and critical cases, and Lung Cleansing and Detoxifying Decoction (LCDD) for all stages (Figure 1B) (China, 2020a; Wang et al., 2020). In addition, patients may be prescribed supplementary herbal medicines based on their individual condition. This strategy of treatment, based on the specific stage of disease progression, demonstrates the concept of “precision medicine” or “individualized treatment” and is a strategy that is commonly practiced by TCM practitioners.

The “3 drugs” in 3-drugs-3-formulas are JQG, LQC, and XI. These drugs were previously approved in China for treating respiratory diseases. The “3 formulas” are LCDD, HD, and XD. These formulas were created to treat COVID-19 by combining several classical formulas and adding complementary herbs. All of the recommended formulas are comprised of traditional formulas that have been used to treat pulmonary and respiratory diseases in China for thousands of years. A summary of disease progression and usage of recommended treatment are shown in Figure1B.

## All five Oral Formulas (Drugs or Decoctions) Have More than One Herb Belonging to the Category of “Qing-Re Herbs”- Known for Their Anti-inflammatory Properties

Zhang, et al. executed *in silico* screen to identify Chinese medical herbs that contain compounds that might directly inhibit SARS-CoV-2. Interestingly, All five oral formulas contain herbs that had anti-SARS-CoV-2 activity as has been suggested by Zhang, et al. (Zhang et al., 2020).

We have developed the STAR (Signal Transduction, Activity and Response) Drug Discovery Platform—a rich database of 300 commonly used medicinal herbs, tested across more than 30 signaling pathways using luciferase reporter assays, enzymes, and other bioassays—now licensed to Yiviva, Inc. Using STAR, we examined the activities of the herb(s) used in the “3-drugs-3-formulas” against TNFα, IL-6, IFN-γ, TGFβ, TLR2, TLR4 pathways, COX-2 and iNOS enzyme activities as well as against Type III protein secretion of Gram (-) bacteria (Lam et al., 2010; Tsou et al., 2016; Lam et al., 2018). Each of these formulas have herbs that inhibit one or more and cover all of these pathways (Table 1). The biological activities of the herbs could be the potential mechanisms of action for these formula treatments to control COVID-19 disease progression. In our own studies we have found that these formulas inhibit multiple pathways of inflammation, induce NRF2 anti-oxidation, and have anti-fibrosis and anti-bacteria activities. These activities could help to explain the mechanisms of action for each formula in treating COVID-19.

**TABLE 1:** Biological activities of 3-drugs-3-formulas.

Formulas	Inflammation						Innate Immunity		Anti-Oxidation	Fibrosis	Anti-Viral	Anti-Bacteria
	TNFα	IL6	IFNγ	COX2	iNOS	GRE	TLR2	TLR4	NRF2	TGFβ	Direct antiviral	Type III protein secretion
Jinhua Qinggan Granule (JQG)	↓↓↓↓	↓↓↓↓↓	↓↓↓↓↓	↓↓↓↓↓↓	↓↓↓↓	↓↓	↓↑↑↑	↓↑↑↑↑	↑↑↑	↓↓	↑↑↑↑ (Zhang et al., (2020))	↓↓
Lianhua Qingwen Capsule (LQC)	↓↓	↓↓↓	↓↓↓↓	↓↓↓↓↓	↓↓↓↓↓	↓↓	↑↑↑↑	↑↑↑↑↑↑	↑↑↑	↓	↑↑↑↑ (Zhang et al., (2020))	↓↓↓↓
Xuanfeibaidu Decoction (XD)	DK^3^	↓↓	↓↓↓	↓↓↓↓	↓↓↓	↓	↑↑↑↑↑	↑↑↑↑↑	↑	↓↓	↑↑ (Zhang et al., (2020))	↓
Huashibaidu decoction (HD)	↓	↓↓↓↓	↓↓↓↓	↓↓↓	↓↓↓↓↓	↓↓↓↑	↑↑↑	↑↑↑(↓↑)	↑	↓	↑↑↑ (Zhang et al., (2020))	↓
Lung Cleanshing and Detoxifying Decoction (LCDD)	↓	↓↓↓	↓↓↓	↓↓	↓↓↓	↓↓↓↑	↑↑↑↑↑↑↑↑↑↓	↑↑↑↑↑↑↑↑↓	↑	↓↓	↑↑↑↑↑ (Zhang et al., (2020))	↓
Xuebijing Injection (XI)	↓	↓↓	↓↓	↓↓	↓↓↓↓	↓↑	↑↑↑	↑↑(↑↓)↓	DK^1^	↓↓	DK^2^	↓

Not all herbs of formulas were examined. The direction of arrows (↑or ↓) in the table represents known ↑ stimulation or ↓ inhibition activity; each arrow represents an herb that has this activity (for example ↓↓↓, represents three (3) herbs having inhibition activity on a given target, whereas ↓↓↓↓↓ represents five (5) herbs having inhibition activity). (↑↓) represents an herb in the formula have biphasic activity, DK1: indicates negative results when 5 herbs of XI were examined in NRF2 luciferase assay , DK2: indicates negative results when 5 herbs of XI were examined against COVID-19 (Zhang et al., 2020), DK3: indicates negative results when 11 out of 13 herbs in XD were examined in TNFα-NFκB luciferase assay and 2 out of 13 herbs have not yet been examined in our lab. Direct antiviral was done by others (Zhang et al., 2020). COX-2 and iNOS are enzymatic reactions. Gram (-) bacteria Type III protein secretion results were published (Tsou et al., 2016). Methods to determine effects of herbs on luciferase activity of reporter cells induced by their corresponding ligands: TNFα- NFκB, IL6-STAT3, IFNγ-STAT1, TGFβ-SMAD2/3, LPS-TLR4- NFκB, PGN-TLR2- NFκB, or COX2/iNOS activities were shown in previous reports (Lam et al., 2018; Lam et al., 2010).

To better understand each of these formulas from the perspective of Chinese medicine, we dissect the components of each. We categorize the components by their TCM classification. A summary of the components of the 3-drugs-3-formulas is shown in Table 2.

**TABLE 2:** Compositions of 3-drugs-3-formulas.

Treatment	Stage	Key Formulation	Additional Formulations	Additional Herbs	Number of Herbs
Jinhua Qinggan Granule (JQG)	Mild	Maxingshigan Decoction • *Ephedra sinica* Stapf.^a^1, Stir-fried *Prunus armeniaca*.^b^2, Gypsum Fibrosum, *Glycyrrhiza glabra* L.3	Yinqiao Poder • *Arctium lappa* L.^b^2, *Lonicera japonica Thunb.*4, *Forshythia suspensa* (Thunb.) Vahl.2, *Mentha canadensis*L.12, *Glycyrrhiza glabra* L.3	*Fritillaria thunbergii* Miq.^b^5, *Scutellaria baicalensis* Georgi, *Anemarrhena asphodeloides* Bunge.8, *Artemisia annua* L.6	12 (1^a^+3^b^+8)
Lianhua Qingwen Capsule (LQC)	Mild	Maxingshigan Decoction • *Ephedra sinica* Stapf.^a^1, Stir-fried *Prunus armeniaca*.^b^2, Gypsum Fibrosum, *Glycyrrhiza glabra* L.3	Yinqiao Powder • *Lonicera japonica* Tgunb. 4, *Forsythia suspensa*(Thunb.) Vahl. 2, l-Menthol, *Glycyrrhiza glabra* .3	*Isatis tinctoria* L.7, *Dryopteris crassirhizoma* Nakai.8, *Rheum palmatum* L.8, *Houttuynia cordata* Thunb.6, *Pogostemon cablin* (Blanco) Benth. 12, *Rhodiola crenulata*(Hook.f. and Thomson) H.Ohba 3	13 (1^a^+1^b^+9+1+1)
Xuanfeibaidu Decoction (HD)	Moderate	Maxingshigan Decoction • *Ephedra sinica* Stapf.^a^1, Stir-fried *Prunus armeniaca*.^b^2, Gypsum Fibrosum, *Glycyrrhiza glabra* L.3	Maxingyigan Decoction • *Ephedra sinica* Stapf.^a^1, Armeniacae Semen Amarum^b^2, *Glycyrrhiza glabra*L.3, *Coix lacryma-jobi var. ma-yuen*(Rom.Caill) Stapf.2,	*Artemisia annua* L.6, *Reynoutria japonica* Houtt.3, Verbena officinalis L.12, *Atractylodes lancea*(Thunb.) DC.8, *Pogostemon cablin* (Blanco) Benth.12, *Citrus × aurantium* L.13	13 (1^a^+2^b^+5+4+1)
			Qianjinweijing Decoction • *Phragmites austalis* subsp. *australis.*8		
			Tinglidazao Xiefei Decoction• *Descurainia sophia* (L.) Webb ex Prantl.^b^2		
Huashibaudu Decoction (HD)	Severe	Maxingshigan Decoction • *Ephedra sinica* Stapf.^a^1, Stir-fried *Prunus armeniaca*.^b^2, Gypsum Fibrosum, *Glycyrrhiza glabra* L.3	Huopoxiefei Decoction• Gingered *Pinellia ternata* (Thunb.) Makini.^b^9, *Pogostemon cablin* (Blanco) Benth.12. Poria cocos (Schw.) Wolf. *Magnolia officinalis* Rehder and E.H. Wilson15,	*Descurainia sophia* (L.) Webb ex Prantl.^b^2,*Rheum palmatum* L.8, *Atractylodes lancea* (Thunb.) DC.8, *Lanxangia tsao-ko*(Crevost and Lemarié) M.F.Newman and Skornick.2, *Paeonia Lactiflora* Pall.7, *Astragalus mongholicus* Bunge,7	14 (1^a^+3^b^+3+4+1+1+1)
Lung Cleansing and Detoxifying Decoction (LCDD)	All stages	Maxingshigan Decoction • *Ephedra sinica* Stapf.^a^1, Stir-fried *Prunus armeniaca*.^b^2, Gypsum Fibrosum, *Glycyrrhiza glabra* L.3	Sheganmahuang Decoction• *Ephedra sinica IStapf.* ^a^ *1, Asarum sieboldii* Miq.^a^3, *Iris domestica*(L.) Goldblatt and Mabb.^b^8, *Aster tataricus* L.f.^b^3, *Tussilago farfara* L.^b^4	*Pogostemon cablin* (Blanco) Benth.12, *Dioscorea polystachya* Turcz.9	21 (5^a^+2^b^+2+5+2+2)
			Xiaochaihu Decoction• *Bupleurum chinese* DC.^a^, *Zingiber officinale* Rosco.^a^, Gingered *Pinellia ternata*(Thunb.) Makino.^b^9, *Scutellaria baicalensis* Georgi7, Honeyed *Glycyrrhiza glabra* L.3		
			Wuling Powder• *Cinnamomum cassia*(L.)J.Presl.^a^11, Poria cocos (Schw.) Wolf, *Polyporus umbellatus* (Pers.) Fries, *Alisma plantago-aquatica*L.9, *Attractylodes macrocephala* Koidz.8,		
			Juzhihiang Decoction• *Zingiber officinale* Rosco.^a^8, *Citrus* × *aurantium*L.13, *Citrus trifoliata*L.2		
Xuebijing (XI)	Severe or critical			*Carthamus tinctorius*L.14, Paeonia lactiflora Pall.7, *Conioselinum anthriscoides* 'Chuanxiong'.8, *Salvia miltiorrhiza* Bunge.7, *Angilica sinensis*(Oliv.) Diels. 7	5

The stages of coronavirus disease-19 are defined by the diagnosis and treatment (8th trial edition) in China (China, 2020a). The unique symbols or text colors correspond to different categories of herbs used in the formulations; ^a^: “Jie-Biao” herbs; ^b^: “Huatan-Zhike-Pingchuan” herbs; green: “Qing-Re” herbs; orange: “Qu-Shi ” herbs; red: “Huo-Xue” herbs; blue: “Bu-Yi” herbs; purple: “Xing-Qi” herbs; some herbs in the table are repeated. Numbers represent medicinal parts of Chinese medicines in the table; 1: herbaceous stem; 2: fruit; 3: root and rhizome; 4: bud; 5: bulb; 6: aerial parts; 7: root; 8: rhizome; 9: tuber; 10: sclerotium; 11: twig; 12: whole herb; 13: peel; 14: flower; 15: bark.

In TCM, herbs categorized as “Qing-Re” (translated as “remove heat”) are used when “heat” symptoms, which cover current inflammation symptoms, have been diagnosed. With the exception of Xuebijing Injection (XI), the five oral formulas in the “3-drugs-3-formulas” all contain “Qing-Re” herbs. Eight of 12 herbs in JQG are “Qing-Re” herbs, nine of 13 herbs in LQC, five of 13 herbs in XD, three of 14 herbs in HD, and two of 21 herbs in LCDD. We previously demonstrated that “Qing-Re” herbs often have multiple mechanisms of anti-inflammatory activity, but 80% of all herbs examined also have one to two actions against six inflammatory mechanisms studied (Guan et al., 2018). Although XI does not contain any “Qing-Re” herbs and all five of its herbs belong to the category of “Huo-Xue” herbs, some of the “Huo-Xue” herbs within XI also promote one or more anti-inflammatory activities (Yu et al., 2013). Thus, the five herbs administered together could potentiate anti-inflammatory activity that may be more powerful than any single herb in the formula. For example, LCDD contains two herbs belonging to “Qing-Re” herbs and 19 herbs belonging to other categories. Together, LCDD exhibits anti-inflammatory activities on all six key inflammatory mechanisms (Table 1). Thus, the common mechanism for all six “3-drug-3-formulas” targets the inflammatory process. The anti-inflammatory activity of these formulas should be considered holistically and sequestered to individual herbs.

As the COVID-19 disease progresses and new symptoms emerge, additional categories of medicinal herbs have been added to the base formulas to improve their efficacy. JQG and LQC are commonly used for mild stages and the majority of herbs used in their formulas are “Qing-Re” herbs. In moderate stages of COVID-19, herbs categorized as “Qu-Shi” translated as “remove dampness have also been included to treat symptoms such as excess mucus secretion and edema, which can cause shortness of breath and hypoxia in patients. Interestingly, many “dampness” symptoms diagnosed by practitioners of Chinese medicine are similar to those of RBS in the advanced pulmonary stage of COVID-19 (Garvin et al., 2020). Studies have suggested that COVID-19 patients requiring time in an intensive care unit (ICU) tend to present severe hypercoagulability along with a severe inflammatory state. Moreover, fibrin formation and polymerization may predispose patients to thrombosis and may correlate with a worse patient outcome (Panigada et al., 2020; Spiezia et al., 2020). Giuseppe et al. also suggested that controlling coagulation disorder may be the key to lowering mortality rates (Magro, 2020). In severe and critical stages, “Huo-Xue” herbs, translated as “activate blood” and “Bu-Yi” herbs, translated as “tonics” are added to the formulas. In TCM, Huo-Xue herbs are used to improve blood circulation, local hypoxia, blood rheology and coagulation; increase local blood flow; promote fibrinolysis, anticoagulation, and antithrombotic activity; eliminate microcirculation obstacles; and inhibit platelet activity (Yu et al., 2013). “Huo-Xue” herbs could be useful in limiting the degree of hypercoagulability and may improve patient outcome for those in severe or critical COVID-19 stages. “Huo-Xue” herbs are used in LQC and HD and injectable XI. “Qing-Re” and “Qu-Shi” herbs are key categories of herbs used in the other three oral formulas to treat patients in moderate to critical stages. “Bu-Yi” herbs are claimed to be useful in improving the physical state of individuals such as fatigue— a major symptom of patients in the severe and critical stages (Yang et al., 2019). Additionally, another category of herbs, “Xing-Qi” herbs, are claimed to “promote the circulation of *qi*” and treat indigestion and loss of appetite. “Bu-Yi” and “Xing-Qi” herbs were added together into the formula of HD and LCDD for treating severe stage patients. The potential synergetic action of these two categories of herb could be interesting to explore.

## All five Oral Formulas Share Maxingshigan Decoction (MD) as a Common Core Formula

With the exception of the injected treatment formula, all five oral treatments (drugs or decoctions) consist of MD. MD is a classic formula described 1800 years ago in *Treatise on Febrile Caused by Cold and Miscellaneous Diseases* (Shang Han Za Bing Lun), a book that introduced the pathogenesis and treatment of infectious diseases in ancient times. The formula consists of four traditional Chinese medicines, *Ephedra sinica* Stapf., herbaceous stem, *Prunus armeniaca* L., fruit, stir-fried, gypsum fibrosum (Shi Gao) and *Glycyrrhiza glabra* L., root and rhizome, raw or honeyed. Historically, MD was used to treat febrile diseases with symptoms of perspiration, panting with no fever, or mild fever. In addition to being used to treat diseases, MD is used today by clinicians to control radio-chemotherapy induced lung injury, acute lung injury, asthma, influenza infection, viral pneumonia, and severe community-acquired pneumonia (Lin, 2015; Li L. et al., 2018; Song et al., 2018; Li, 2020a; Zhou X. et al., 2020). *Ephedra sinica* Stapf., herbaceous stem has been claimed to be effective for allaying asthma and for inducing diaphoresis and diuresis, but it could have severe adverse effects (Commission, 2010). *Glycyrrhiza glabra* L., root and rhizome, raw or honeyed (GG) is included in the formula as it is claimed to decrease adverse effects of *Ephedra sinica* Stapf., herbaceous stem (Wei et al., 2016a; Wei et al., 2016b). GG has been used for centuries in TCM for the treatment of cough and influenza virus (Lin et al., 2016). Flavanone liquiritigenin and its precursor and isomer chalcone isoliquiritigenin are the main bioactive constituents of GG, and have also been suggested to have anti-inflammatory activities (Ramalingam et al., 2018). In addition, when used in silico screening, GG was claimed to have the potential to directly inhibit SARS-CoV-2 (Zhang et al., 2020). *Prunus armeniaca* L., fruit, stir-fried is used as an anti-asthmatic, a mucolytic, an expectorant, and a laxative agent. Amygdalin, a cyanogenic diglucoside found in *Prunus armeniaca* L., fruit, stir-fried, could be metabolized in the human body to produce hydrocyanic acid which could inhibit the respiratory center in the brain to render smoother breathing, thereby gradually reducing cough and asthma (Shi and Liu, 2018). Gypsum fibrosum is mainly composed of calcium sulfate dihydrate, with the chemical formula CaSO_4_⋅2H_2_O. It is used to treat febrile diseases, dysphoria and thirst in TCM practice. A study indicated that gypsum fibrosum exerted anti-inflammatory effects, but the mechanisms are still unclear (Yin, 2019). MD has been suggested to be able to regulate viral infection and immune inflammatory response by promoting TH17 cell differentiation and T-cell homeostatic proliferation to inhibit virus proliferation, and to negatively regulate immune inflammatory factors such as interleukin, TNFα and integrin, thus decreasing the degree of cytokine storm in patients with COVID-19 (Zhang et al., 2020). Zhang et al. (Zhang et al., 2013) also found that MD could significantly reduce the inflammatory response in lungs of mice infected with influenza virus. The mechanism may be related to the inhibition of neuraminidase activity and prevention of viral proliferation. MD not only has anti-inflammatory and antiviral effects, but it is also known for its antitussive and antipyretic properties (Lin et al., 2016) which could alleviate cough or fever symptoms in COVID-19 patients. Recently Li, et al. suggested that MD might directly inhibit the absorption and replication of SARS-CoV-2, prevent cytokine storm and relieve lung injury. However, the difference of spectrum of actions between MD and the other four formulas based on MD wasn’t mentioned (Li et al., 2020c). This formula of MD alone may not be sufficient for treating COVID-19, but it appears to be the core formula in the five oral formulas of the “3-drugs-3-formulas”. It should be noted that *Ephedra sinica* Stapf., herbaceous stem is banned in several regions of the world due to its severe adverse effects. Low-dose *Ephedra sinica* Stapf., herbaceous stem extracts can reduce body weight and can improve athletes’ physical performance. However, high doses or long-term use of Ephedra extracts can cause various adverse effects (Gardner et al., 2003; Worley and Lindbloom, 2003; Miao et al., 2020). Consequently, the formulas containing *Ephedra sinica* Stapf., herbaceous stem will need to overcome regulatory hurdles in order to be used in countries where its usage is heavily regulated. At relevant dosage, MD may not have the adverse effects listed above that are attributed to *Ephedra sinica* Stapf., herbaceous stem, due to the proportionally low concentration of *Ephedra sinica* Stapf., herbaceous stem used relative to the other herb components. Nevertheless, the adverse effects of *Ephedra sinica* Stapf., herbaceous stem should be further studied in light of its potential benefits.

## For Treatment of the Mild Stage of COVID-19 - Jinhua Qinggan Granule (JQG) and Lianhua Qingwen Capsule (LQC)

Both JQG and LQC are approved drugs in China. Their formulas share MD, as well as another common TCM formula: Yinqiao Powder (YP), which was first described in the *Treatise on Differentiation and Treatment of Epidemic Febrile Disease* (Wen Bing Tiao Bian) published in 1798. Historically, YP was used for treating early stages of epidemic febrile diseases with symptoms of aversion to “heat”, however, not to treat “cold” and “thirst” symptoms. In modern times, YP is mainly used to treat upper respiratory diseases, laryngopharyngeal inflammation, suppurative tonsillitis, and viral infection. An *in vivo* study suggested that YP could inhibit the release of pro-inflammatory cytokines of IL-1β and TNFα in the early stage of sepsis (He et al., 2016). Lei et al. suggested that YP may boost the mucosal immune system to prevent and treat upper respiratory diseases by improving lysozymal activity and by increasing SIgA levels in saliva (Lei et al., 2013). Additionally, its components, *Lonicera japonica* Thunb., bud and *Forsythia suspensa* (Thunb.) Vahl., fruit, were suggested to have direct inhibitory potential on SARS-CoV-2 and on anti-inflammatory activities (Guo et al., 2015; Zhang et al., 2020). YP is widely used for preventing and treating upper respiratory diseases. Ultimately, YP could enhance the anti-inflammatory and antiviral activities of MD.

The key differences in herbal components of JQG and LQC are as follows. The drug formula JQC has additional “Qing-Re” herbs *Scutellaria baicalensis* Georgi., root, *Anemarrhena asphodeloides* Bunge., rhizome, *Artemisia annua* L.*,* aerial parts; “Jie-Biao” herbs *Arctium lappa* L., fruit; “Huatan-Zhike-Pingchuan” herbs *Fritillaria thunbergii* Miq., bulb; while the drug formula LQC, adds “Qing-Re” herbs *Isatis tinctoria* L., root, *Dryopteris crassirhizoma* Nakai., rhizome, *Rheum palmatum* L., rhizome, *Houttuynia cordata* Thunb., aerial parts; “Qu-Shi” herbs *Pogostemon cablin* (Blanco) Benth*.,* whole herb, “Huo-Xue” herbs *Rhodiola crenulata* (Hook.f. and Thomson) H. Ohba., root and rhizome.

JQC consists mainly of three categories of herbs: “Jie-Biao” herbs, “Huatan-Zhike-Pingchuan” herbs and “Qing-Re” herbs. LQC includes additional “Qu-Shi” herbs and “Huo-Xue” herbs to enhance anti-inflammatory activities and to improve symptoms related to “dampness” such as excessive mucus secretion, edema or “yellow-greasy coating of tongue” in COVID-19 patients. Moreover, LQC is better at resolving fever. Compared to LQC, JQG has a stronger antitussive effect due to its use of *Fritillaria thunbergii* Miq., bulb and *Arctium lappa* L., fruit, which are herbs commonly used for pharyngeal diseases to improve expectoration and to relieve cough and sore throat. In summary, JQG and LQC are two oral drugs that are used for treating patients who fall under the mild state of COVID-19. Duan, et al. (Duan et al., 2020) suggested that JQC could significantly reduce fever time, alleviate clinical symptoms of cough, fatigue and expectoration, and relieve psychological anxiety in patients with mild COVID-19. Hu, et al. (Hu et al., 2020) conducted a multicenter, prospective, randomized controlled trial (RCT) upon the efficacy and safety of Lianhua Qingwen capsules involving 284 patients randomized to receive usual treatment alone or in combination with LQC. Results suggested that LQC could be considered to ameliorate clinical symptoms of Covid-19 including shortening recovery of fever, fatigue and coughing. These two drugs share two common classic formulas, MD and YP, as well as different complementary herbs. So, each formula has its own advantage. JQG more effectively treats COVID-19 patients with a cough, likely due to the presence of *Fritillaria thunbergii* Miq., bulb and *Arctium lappa* L., fruit*,* while LQC is used to treat patients with fevers, likely due to the presence of additional “Qing-Re” herbs.

## For Treatment of the Moderate Stage of COVID-19-Xuanfeibaidu Decoction (XD)

XD is composed of MD, plus three additional traditional formulas (Maxingyigan Decoction, Qianjinweijing Decoction, Tinglidazao Xiefei Decoction), and six additional herbs. It is suitable for the treatment of moderate stages of COVID-19, including symptoms of “dampness-toxin stagnating in the lung syndrome”. Clinical manifestations include fever, cough with little sputum or yellow sputum, airway obstruction and short of breath, abdominal distension and inhibited defecation, “dark-red plump tongue with yellow greasy or yellow dry coating”, and “slippery rapid or wiry slippery pulse” (China, 2020a). Five of 13 herbs in XD are “Qing-Re” herb and four of 13 are “Qu-Shi” herbs. “Qu-Shi” herbs are often used for treating mucus secretion, sputum, and edema. According to China’s COVID-19 guidelines (China, 2020a), abnormal chest imaging starts to present in moderate stage cases, indicating disease progression spreading of the lesion from the upper respiratory tract to the lungs. A series of studies (Liu and Liang, 2014; Li et al., 2019; Ma et al., 2020) suggest that XD component herbs, *Coix lacryma-jobi var. ma-yuen (Rom.Caill.)* Stapf., fruit, *Phragmites australis* subsp. *australis*, rhizome, *Atractylodes lancea* (Thunb.) DC*.,* rhizome and *Pogostemon cablin* (Blanco) Benth*.,* whole herb, all promote anti-inflammatory activity. Moreover, *Coix lacryma-jobi var. ma-yuen (Rom.Caill.)* Stapf., fruit and *Pogostemon cablin* (Blanco) Benth*.,* whole herb have been suggested to have analgesic, immune enhancement, and anti-microbial properties. A forty-two patients randomized clinical trial suggested that XD combined with conventional medicine may significantly improve patient’s clinical symptoms, increase the number of white blood cells and lymphocytes to improve immunity, and also significantly reduce C-reactive protein and erythrocyte sedimentation rate to exert anti-inflammatory effect (Xiong et al., 2020). Network pharmacology and molecular docking studies have revealed that XD may inhibit viral invasion and viral replication by binding to ACE2 receptors and to 3CLPro of SARS-CoV-2 through its flavonoids and phytosterols. XD may have direct antiviral activities. Additionally, *Descurainia sophia* (L.) Webb ex Prantl, fruit, a component of XD has also been suggested to have direct anti-coronavirus effects (Zhang et al., 2020). Moreover, XD may play a role in the treatment of COVID-19 by regulating key targets such as MAPK3, MAPK1, CCL2, EGFR, and NOS2 after viral infection of cells, exerting an anti-cytokine storm against IL-6 and IL-1β, having an anti-oxidation effect, and regulating the body's immunity. In summary, XD includes more herbs, in addition to MD, that promote anti-inflammatory and immune enhancing activities, ultimately enhancing the anti-inflammatory properties of the XD formula to treat moderate cases of COVID-19. In addition, ACE2 is also involved in the bradykinin storm pathway. The XD formula has been claimed to relieve dampness symptoms which mimic RBS symptoms (Guo, 2020). Thus, the XD formula may also interact with ACE2 or COX-2 as well as NF-κB pathways which could relieve RBS. This should be further explored.

## For Treatment of Severe Stage of COVID-19—Huashibaidu Decoction

HD is composed of MD, Huopoxialing Decoction, *Descurainia sophia* (L.) Webb ex Prantl., fruit-also in XD, *Paeonia lactiflora* Pall., root, *Rheum palmatum* L., rhizome*, Astragalus mongholicus* Bunge., root, *Atractylodes lancea* (Thunb.) DC., rhizoma, *Lanxangia tsao-ko* (Crevost and Lemarié) M.F.Newman and Skornick.*,* fruit. HD is recommended for treating COVID-19 patients with shortness of breath. Clinical manifestations include “fever with flushed face”, “cough with little yellow sticky sputum, or blood-stained sputum”, airway obstruction and shortness of breath, lassitude, “dryness, bitterness, and stickiness in the mouth”, nausea and loss of appetite, inhibited defecation, “scanty dark urine”, “red tongue with white greasy coating”, and “slippery rapid pulse” (China, 2020a). Intriguingly, HD (as well as XD) includes additional “Qing-Re” herb: *Rheum palmatum* L., rhizome, raw, “Huo-Xue” herb: *Paeonia lactiflora* Pall., root. and “Bu-Yi” herb: *Astragalus mongholicus* Bunge., root. *Descurainia sophia* (L.) Webb ex Prantl., fruit, *Astragalus mongholicus* Bunge., root. and *Glycyrrhiza glabra* L., root and rhizome, raw or honeyed were also suggested to have direct anti-coronavirus effects (Zhang et al., 2020). Its composite Huopoxialing Decoction is a formula used in TCM for its strong ability to “resolve dampness”. HD’s formula appears to be designed to resolve symptoms in severe COVID-19 patients such as a cough with an increase in sputum secretion, airway obstruction or shortness of breath, and fatigue. In TCM, the manifestations of “dampness” appear to be related to excess interstitial fluid accumulation, such as mucus secretion and edema. A pathological investigation of two severe COVID-19 patients, indicated excessive mucus secretion with serous and fibrinous exudation. The exudation could aggravate the dysfunction of ventilation and may be one of the pathogenic mechanisms responsible for hypoxemia (Wang et al., 2020b). At this stage, symptoms appear to mimic those associated with RBS-pulmonary edema, shortness of breath, and gastrointestinal (GI) disorders, all of which fall under the classification of “dampness” syndrome. One RCT (ChiCTR2000030988) with sample size of 204 evaluated the effectiveness of HD comparing with Western medicine. Three additional studies on HD had been carried out in Jinyintan Hospital (75 severe cases), Dongxihu Fangcang Hospital (124 moderate cases), and Jiangjunlu Street Health Center (894 mild and moderate cases), respectively (Luo et al., 2020b). The results suggested significant improvement in symptoms and CT images of lungs, shortening hospital stay and reducing rate of viral clearance by polymerase chain reaction, and no adverse events or liver and kidney damage were found (China, 2020b). Network pharmacology studies and molecular docking analyses have demonstrated that signaling pathways involving HD include TNFα, PI3K–Akt, NOD-like receptor, MAPK, and HIF-1. Baicalein and quercetin are the top two compounds of HD, with high affinity for ACE2 (Tao et al., 2020). Another study has suggested that quercetin not only impairs the binding of viral S-protein to ACE2 receptor, but also has virus neutralizing effect of quercetin on SARS-CoV-2 (Pan et al., 2020). As mentioned, ACE2 is a key component of the bradykinin storm in addition to serving as a receptor of SAR-CoV-2. Baicalein and quercetin are major compounds of *Scutellaria baicalensis* Georgi., root, a key herb in JQG and LCDD formulas. In summary, MD is the core formula of HD, Huopoxialing Decoction is included to “resolve dampness”, and additional herbs eliminate or relieve symptoms of cough with sputum secretion, airway obstruction, short of breath, and fatigue in severe COVID-19 cases. The impact of the HD formula in relieving bradykinin storm should be further studied.

## For Treatment of all stages—Lung Cleansing and Detoxifying Decoction (LCDD)

LCDD was claimed to have excellent efficacy in the clinic by National Administration of Traditional Chinese Medicine of China and has been recommended as a universal formula to treat all stages of COVID-19 in China (China, 2020a). It has been shown to relieve cough, shortness of breath, and panting in COVID-19 patients. An *in vivo* study suggested that Xiaochaihu Decotion, decreased TNFα, IL-1β, and IL-6 level in plasma of LPS-induced inflammation mice. This indicates that Xiaochaihu Decoction has anti-inflammatory properties. *Bupleurum chinense* DC., root and *Scutellaria baicalensis* Georgi., root, two components of Xiaochaihu Decoction, contain flavonoids that are known to be effective antiviral agents (Zakaryan et al., 2017). Moreover, polysaccharides extracted from Bupleurum have been suggested to have an immunomodulatory effect via the NF-κB signaling pathway (Song et al., 2017). Another component formula, Wuling Powder, is a classic formula known for “resolving dampness” by promoting urination. Modern pharmacological research also suggests that Wuling Powder can eliminate edema, improve gastrointestinal function, remove free radicals, reduce lipid peroxide levels, increase superoxide dismutase activity, reduce the production of inflammatory cytokines, and regulate the body immune function, etc. (Yao et al., 2020). Therefore, Wuling Powder may improve lung tissue edema, protect gastrointestinal function, and have anti-oxidation and anti-inflammatory effects, reducing COVID-19 damage to target tissues, and regulating immunity to improve the body's antiviral ability to facilitate disease recovery. In another component formula, Juzhijiang Decoction, *Citrus × aurantium* L., peel has antithrombotic and anti-inflammatory actions; *Citrus trifoliata* L. fruit may improve myocardial metabolism and also has anti-inflammatory activity (Yao et al., 2020). Two additional herbs, *Pogostemon cablin* (Blanco) Benth*.,* whole herb and *Dioscorea polystachya* Turcz., tuber. *Dioscorea polystachya* Turcz., tuber are included to relieve gastrointestinal symptoms in COVID-19 patients, such as abdominal distention, loss of appetite, and diarrhea. *Glycyrrhiza glabra* L., root and rhizome, raw or honeyed and *Dioscorea polystachya* Turcz., tuber belong to “Bu-Yi” herbs in TCM and are used to treat patients with fatigue – one of the main symptoms in COVID-19 patients (Li et al., 2020b), known to worsen throughout COVID-19 disease progression and to hinder patient recovery. In summary, in addition to reducing lung damage, LCDD may reduce other tissue damage, such as in the kidney, liver, heart and brain. Components of LCDD, *Aster tataricus* L. f., root and rhizome*, Tussilago farfara* L., bud*, Bupleurum chinense* DC., root*, Glycyrrhiza glabra* L., root and rhizome, raw or honeyed, may have direct anti-SARS-CoV-2 effects (Zhang et al., 2020). Six registered trials evaluated the effect of LCDD: One (ChiCTR2000030810) was a registered RCT comparing LCDD versus Western medicine with sample size of 100; one (ChiCTR2000029778) was a controlled clinical trial comparing LCDD plus Western medicine versus Western medicine only with sample size of 600; Three (ChiCTR2000030864, ChiCTR2000030883, and ChiCTR2000032767) Were single-arm studies; one (ChiCTR2000030806) was a retrospective study evaluating the effectiveness of LCDD plus ulinastatin, a human urinary trypsin inhibitor. A controlled clinical trial suggested that the combination of LCDD with Western medicine demonstrated effects of anti-inflammatory and mitigating the extent of multi-organ impairment compared with those of Western medicine alone in patients with mild and moderate COVID-19 (Xin et al., 2020).

LCDD builds upon MD with four additional component formulas to enhance the anti-inflammatory activity of MD and to relieve symptoms occurring in the cardiovascular system, gastrointestinal system, or kidneys, which are out of the typical therapeutic scope of MD and are essential in fulfilling the clinical needs of COVID-19 patients. *Asarum sieboldii* Miq., root and rhizome, gingered is also a restricted herb in the United States, Germany, and other regions. *Asarum sieboldii* Miq., root and rhizome, gingered contains aristolochic acid which may be carcinogenic, mutagenic, and/or nephrotoxic (Han et al., 2019). Thus, it is necessary to study the importance of *Asarum sieboldii* Miq., root and rhizome, gingered action as a component of of LCDD to allow most efficient use. We compared LCDD, with and without *Asarum sieboldii* Miq., root and rhizome, gingered, using the LPS-induced inflammation model of mice. Our preliminary results clearly indicated that the full formula of LCDD better protects lung and systemic inflammation than LCDD without *Asarum sieboldii* Miq., root and rhizome, gingered(data not shown). This suggests that *Asarum sieboldii* Miq., root and rhizome, gingered. is an essential component of LCDD action and confirms the challenge that will exist in bringing LCDD to the market in many countries. Further studies will be required to demonstrate the effects of LCDD used at the clinically relevant dosage and to further look at the carcinogenic, mutagenic, and nephrotoxic characteristics of *Asarum sieboldii* Miq., root and rhizome, gingered.

## For Treatment of Severe and Critical COVID-19 stages—Xuebijing Injection (XI)

Xuebijing Injection (XI) is the only injectable formula among the 3-drugs-3-formulas. A published meta-analysis of the efficacy and safety of XI combined with conventional treatment of sepsis (Li C. et al., 2018), including 16 high-quality randomized controlled trials (Jadad score ≥3 points), a total of 1,144 sepsis patients showed that compared with conventional treatment, combined XI could reduce 28-days mortality, APACHE Ⅱ score, body temperature, white blood cell count and other indicators of sepsis patients, and no obvious adverse reactions. XI is composed of five herbs (Table 2), all of which belong to the “Huo-Xue” category of herbs. In TCM, “Hue-Xue” herbs are used to treat traumatic injury, menstrual disorders, amenorrhea, dysmenorrhea, rheumatism caused pain, cardiovascular disease, etc. “Huo-Xue” herbs have been claimed to have anti-inflammatory, antibacterial, analgesia, antitumor, cardiovascular regulatory, anti-asthmatic, anti-myocardial ischemia and antithrombotic properties (Yu et al., 2013). Many patients with severe cases of COVID-19 present coagulation abnormalities that mimic other systemic coagulopathies associated with severe infections, such as disseminated intravascular coagulation (DIC) or thrombotic microangiopathy (Levi and Scully, 2018). Mounting studies have revealed that abnormal coagulation in severe COVID-19 cases is associated with a poor prognosis and increased risk of death (Tang et al., 2020a; Tang et al., 2020b). “Huo-Xue” herbs have shown substantial antithrombotic effects (Yu et al., 2013); however, the mechanisms of action may differ depending on the herb used from the category. “Huo-Xue” herbs may decrease risk of death in severe and critical COVID-19 patients with abnormal coagulation by anti-myocardial ischemia, antithrombotic actions, etc. It should be noted that some of the herbs used in XI such as *Rhodiola crenulata* (Hook.f. and Thomson) H. Ohba., root and rhizome are components of LQC. *Paeonia lactiflora* Pall., root is also in HD. The capabilities of the XI formula could also include its potential anti-viral, anti-inflammatory, and anti-RBS properties.

## Conclusion and Perspectives

While China was once the epicenter of COVID-19, it has essentially contained the outbreak of COVID-19. It is highly likely that TCM played a pivotal role in treating COVID-19 patients and thus in containing the outbreak. The 3-drugs (JQC, LQC and XI) are established formula drugs in China that have been used for the treatment of respiratory diseases; the 3-formulas (LCDD, HD and XD) are new formulas that evolved by the combining different formulas to treat different stages of COVID-19. The five oral formulas JQG, LQC, LCDD, HD and XD all build upon the core formula of Maxingshigan Decoction (MD). While MD has anti-inflammatory activities, it is likely not sufficient to serve as a standalone treatment. Thus, additional formulas or herbs with unique activities have been added to increase anti-inflammatory activity and to relieve other symptoms caused by infection. “Qing-Re” herbs play a key anti-inflammatory role, while the four other herbal categories, “Qu-Shi”, “Huo-Xue”, “Bu-Yi”, and “Xing-Qi” herbs are included to treat symptoms such as excessive mucus secretion, edema, coagulation, fatigue, loss of appetite and indigestion, etc. It should be noted that *Ephedra sinica* Stapf., herbaceous stem and *Asarum sieboldii* Miq., root and rhizome, gingered, two “Jie-Biao” herbs in the six formulas, can cause a decrease in symptoms of high fever and can also cause weight-loss (Worley and Lindbloom, 2003; Perwaiz, 2014). Whether most of “Jie-Biao” herbs share similar metabolic properties should be explored. Among the six formulas, only HD and LCDD, the two formulas used to treat severe stages of COVID-19, utilize “Bu-Yi” herbs to resolve fatigue. The usage of different categories of herbs depend on the severity of disease progression. Some herbs may have a direct or indirect antiviral effect. Herb components with a direct antiviral effect may target ACE2, the SARS-CoV-2 receptor and key component of the bradykinin axis, or the serine protease TMPRSS2. An indirect antiviral effect may induce type I interferon (IFN-α and β). It should be noted that in early stages, IFN-α and IFN-β could be critical for innate and adaptive immune responses against SARS-CoV-2. Some of these formulas could also induce IFN-α and β expression (data not shown). However, IFN-α and IFN-β may also facilitate the occurrence of hyper-inflammation and may play a central pathogenic role in severe and critical patients with COVID-19 (Ruscitti et al., 2020). Thus, it is critical that the appropriate drug is chosen for each stage of COVID-19.

Although we focused on discussing “3-drugs-3-formulas”, there are many more formulas and drugs used in the prevention and treatment of COVID-19 in China and beyond (Luo et al., 2020a; Zhao et al., 2020). To expand the use of these treatments beyond China and to the rest of the world, three key steps must be taken. First, rigorous and objective quality control (QC) is needed to ensure consistent, high-quality preparations. Due to the complexity of these formulas, chemical analysis may not be powerful enough to predict biological activity among batches. Our lab has developed an advanced mechanism-based quality control platform (Mech QC) designed to assess the quality and batch-to-batch consistency of complex mixtures focusing biological activity (Lam et al., 2018). Mech QC could be applied to assess and control the consistency of these formulas. The five oral formulas contain, on average, 17 herbs. Efforts should be made to simplify the formulas and to replace the restricted herbs while ensuring the same clinical results. Second, stringent clinical trials are needed. Ideally, multiregional, placebo-controlled, double-blind, randomized clinical studies should be conducted to explore the effects of gender, ethnicity, region, lifestyle, and diet on COVID-19. Moreover, the design of clinical trials is critical. Additional add-on clinical studies would be more suitable for investigating the effects of TCM treatment candidates. Potential adverse effects for each treatment must be investigated. As mentioned previously, Ephedra sinica Stapf., herbaceous stem and Asarum sieboldii Miq., root and rhizome, gingered may have adverse effects at certain dosages. These two herbs have been considered safe when used in certain formulas, where it is possible that either the dosages were low enough to minimize adverse effects, or other herbs in these formulas reduced potential adverse effects. Further studies are needed. In addition, for optimal treatment, one may need to consider the role of circadian rhythms due to circadian clocks’ role in regulating physiology pharmacokinetics and efficacy of many therapeutics (Ruben et al., 2019). Drug interactions should also be carefully monitored. Many of the clinical trials integrating “Western” and Chinese medicine to treat COVID-19 have not been randomized-double blind trials. These trials also lack details about the dosages and categories of Western medicines used, and they do not explain the randomization methodology. In a recent publication, the status of clinical trials using herbal medicines was reported (Zhou Z. et al., 2020). Third, the mechanisms of action and active compounds for each formula need to be further explored. This knowledge would explain how these formulas work, may be helpful in simplifying the formulas, and could improve quality control.

Traditional medicines, including TCM, have evolved with human usage over time and across cultures. The experienced-based nature and systems biology approach of traditional medicines can and should be developed into modern evidence-based medicines to treat current unmet medical needs. The systems biology effect of botanical medicines should be considered a component of the treatment regimen in light of their anti-inflammatory and perhaps anti-RBS and positive cardiovascular impact on different organs. The usage of these formulas could be most effective in combination with reductionist, antiviral, anti-inflammatory, or anti-RBS drugs. Many groups have used network pharmacology to understand the multiple actions of herbs. It should be kept in mind that this approach is only helpful in forming hypotheses; evidence-based studies are still needed to test those hypotheses.

While COVID-19 treatment is critical, prevention will be the best solution in controlling the disease. In China, TCM is utilized in national policies as preventative medicine. More rigorous studies and concrete evidence are needed to validate the efficacy of these preventative formulas and drugs. Given the many environmental, social, and genetic factors that influence our health, now more than ever, our diverse needs necessitate new and different preventative measures. After multiple SARS-CoV-2 strains isolated from different regions were sequenced, research teams from various countries applied the information to begin developing a vaccine. To date, ten coronavirus vaccines have been approved (Craven, 2020). In theory, a vaccine for an RNA virus such as SARS-CoV-2 would be ideal. However, in practice, a single vaccine may not be sufficient given industry experiences developing vaccines for past influenza viruses. The influenza vaccine must be updated yearly to account for viral mutation or draft. One study shows that SARS-CoV-2 could exist in patients who have virus-specific immunoglobulin G (IgG) for an unexpectedly short time (36–50 days). This raises the question of whether patients with antibodies are still at risk for reinfection (Wang et al., 2020a). There are many unknowns regarding the efficacy and long-term adverse effects of a future COVID-19 vaccine and its ability to benefit enough patients for a sustained period of time. Modern botanical drugs could be used to aid in prevention and treatment of COVID-19 and could serve as adjuvant therapy for vaccines. This should be pursued.

COVID-19 patients recovering from viral infection may develop long-term health issues such as fatigue, cardiovascular, neurological, renal, and pulmonary issues (Gan et al., 2020; Gupta et al., 2020). It is important that these long-term risks be taken into consideration during treatment. By nature, Chinese medicine acts on many targets and therefore may be such a solution to address the long-term health risks. Currently, Western and Eastern practices take two fundamentally different approaches to medicine. Western (W) medicine takes a potent, targeted approach with a narrow spectrum of action while Eastern (E) medicine (including TCM) utilizes multiple chemicals, acting on several targets through a broad spectrum, systems biology approach. The treatment and prevention of COVID-19 would benefit greatly from a more effective, integrated, WE Medicine approach in order to address the complexities and pathogenesis of SARS-CoV-2.

## Data Availability

The original contributions presented in the study are included in the article/Supplementary Material, further inquiries can be directed to the corresponding authors.

## References

[B1] Beigel J. H. Tomashek K. M. Dodd L. E. (2020). Remdesivir for the treatment of Covid-19-preliminary report. Reply. N. Engl. J. Med. 383, 994. 10.1056/NEJMc2022236 32649078

[B2] China N. H. C. O. T. P. S. R. O. (2020a). Diagnosis and Treatment of COVID-19 (8th trial edition). Editor China N. H. C.O.T.P.S.R.O. (Beijing: National Health Commission of the People's Republic of China).

[B3] China T. S. C. I. O. O. (2020b). Press conference on the important role of TCM in preventing and treating COVID-19 and effective drugs [Online]. The State Council Information Office of China. Available at: http://www.scio.gov.cn/xwfbh/xwbfbh/wqfbh/42311/42768/index.htm (Accessed November 28, 2020).

[B4] Commission C. P. (2010). Pharmacopoeia of People’s Republic of China. Beijing: China Medical Science Press.

[B5] Craven J. (2020).COVID-19 vaccine tracker. Available at: https://www.raps.org/news-and-articles/news-articles/2020/3/covid-19-vaccine-tracker (Accessed February 15, 2021).

[B79] Duan C. X. Wenguang Z. Chanjuan S. Guobing L. Zhengliang L. Qinglin L. (2020). Clinical observation of Jinhua Qinggan granule in treating pneumonia infected by novel coronavirus. J. Trad. Chin. Med. 61, 1473–1477.

[B6] Farah Yusuf Mohamud M. Garad Mohamed Y. Mohamed Ali A. Ali Adam B. (2020). Loss of taste and smell are common clinical characteristics of patients with COVID-19 in Somalia: a retrospective double Centre study. Infect. Drug Resist. 13, 2631–2635. 10.2147/IDR.S263632 32801800 PMC7406326

[B7] Gan R. Rosoman N. P. Henshaw D. J. E. Noble E. P. Georgius P. Sommerfeld N. (2020). COVID-19 as a viral functional ACE2 deficiency disorder with ACE2 related multi-organ disease. Med. Hypotheses. 144, 110024. 10.1016/j.mehy.2020.110024 32758871 PMC7308773

[B8] Gardner S. F. Franks A. M. Gurley B. J. Haller C. A. Singh B. K. Mehta J. L. (2003). Effect of a multicomponent, ephedra-containing dietary supplement (Metabolife 356) on Holter monitoring and hemostatic parameters in healthy volunteers. Am. J. Cardiol. 91, 1510. 10.1016/s0002-9149(03)00413-2 12804749

[B9] Garvin M. R. Alvarez C. Miller J. I. Prates E. T. Walker A. M. Amos B. K. (2020). A mechanistic model and therapeutic interventions for COVID-19 involving a RAS-mediated bradykinin storm. Available at: https://www.ncbi.nlm.nih.gov/pubmed/32633718 (Accessed. July 7, 2020). 10.7554/eLife.59177 PMC7410499 32633718

[B10] Guan F. Lam W. Hu R. Kim Y. K. Han H. Cheng Y. C. (2018). Majority of Chinese medicine herb category “qing Re Yao” have multiple mechanisms of anti-inflammatory activity. Sci. Rep. 8, 7416. 10.1038/s41598-018-25813-x 29743639 PMC5943244

[B11] Guo C. J. H. L. Y. (2020). Manifestation of strengthening body and dispelling pathogenic factors rule in TCM with “three medicines and three prescriptions”. J. Liaoning Univ. Tradi. Chin. Med. 22, 159–163. 10.13194/j.issn.1673-842x.2020.10.038

[B12] Guo Y. P. Lin L. G. Wang Y. T. (2015). Chemistry and pharmacology of the herb pair Flos Lonicerae japonicae-Forsythiae fructus. Chin. Med. 10, 16. 10.1186/s13020-015-0044-y 26161134 PMC4497423

[B13] Gupta A. Madhavan M. V. Sehgal K. Nair N. Mahajan S. Sehrawat T. S. (2020). Extrapulmonary manifestations of COVID-19. Nat. Med. 26, 1017–1032. 10.1038/s41591-020-0968-3 32651579 PMC11972613

[B14] Han J. Xian Z. Zhang Y. Liu J. Liang A. (2019). Systematic overview of aristolochic acids: nephrotoxicity, Carcinogenicity, and underlying mechanisms. Front. Pharmacol. 10, 648. 10.3389/fphar.2019.00648 31244661 PMC6580798

[B15] He Y. Z. G. Xiao B. He D. He Y. Ai B. (2016). Comparisons of the effects of liangge powder and Yinqiao powder on inflammatory cytokines in early stage of sepsis. J. Hunan Univ. of CM. 36, 7–10. 10.3969/j.issn.1674-070X.2016.12.002

[B16] Hu K. Guan W. J. Bi Y. Zhang W. Li L. Zhang B. (2020). Efficacy and safety of Lianhuaqingwen capsules, a repurposed Chinese herb, in patients with coronavirus disease 2019: a multicenter, prospective, randomized controlled trial, Phytomedicine. 10, 153242. 10.1016/j.phymed.2020.153242 PMC7229744 33867046

[B17] Jin X. Lian J. S. Hu J. H. Gao J. Zheng L. Zhang Y. M. (2020). Epidemiological, clinical and virological characteristics of 74 cases of coronaviru-infected disease 2019 (COVID-19) with gastrointestinal symptoms. Gut. 69, 1002–1009. 10.1136/gutjnl-2020-320926 32213556 PMC7133387

[B18] Jordan R. E. Adab P. Cheng K. K. (2020). Covid-19: risk factors for severe disease and death. BMJ. 368, m1198. 10.1136/bmj.m1198 32217618

[B19] Lam W. Bussom S. Guan F. Jiang Z. Zhang W. Gullen E. A. (2010). The four-herb Chinese medicine PHY906 reduces chemotherapy-induced gastrointestinal toxicity. Sci. Transl. Med. 2, 45ra59. 10.1126/scitranslmed.3001270 20720216

[B20] Lam W. Ren Y. Guan F. Jiang Z. Cheng W. Xu C. H. (2018). Mechanism based quality control (MBQC) of herbal products: a case study YIV-906 (PHY906). Front. Pharmacol. 9, 1324. 10.3389/fphar.2018.01324 30510512 PMC6252377

[B21] Lei N. L. Y. He F. Han C. Lin Q. (2013). Effect of prescription with function of relieving the exterior syndrome on upper respiratory tract infection in mice model through regulating mucosal immunity. Chin. J. Experi. Tradit. Med. Form. 19, 174–177. 10.11653/syfj2013180174

[B22] Levi M. Scully M. (2018). How I treat disseminated intravascular coagulation. Blood. 131, 845–854. 10.1182/blood-2017-10-804096 29255070

[B23] Li C. Wang P. Zhang L. Li M. Lei X. Liu S. (2018). Efficacy and safety of Xuebijing injection (a Chinese patent) for sepsis: a meta-analysis of randomized controlled trials. J. Ethnopharmacol. 224, 512–521. 10.1016/j.jep.2018.05.043 29860133

[B24] Li L. W. Min J. Ouyang J.-J. Hu J. Liao C. Yuan P. (2018). Study on screening and mechanism of effective Chinese medicine for influenza virus pneumonia. Chin. J. Immunology. 34, 1168–1173.

[B25] Li F. Liu X. Yu X. Xu X. Yang H. (2019). Optimization of the extraction, preliminary characterization, and anti-inflammatory activity of crude polysaccharides from the stems of Trapa quadrispinosa. RSC Adv. 9, 22540–22550. 10.1039/C8RA09994D PMC9066627 35519496

[B26] Li J. L. S. Xie Y. Zhao H. Feng Z. Chun L. (2020b). Clinical characteristics and TCM syndrome distribution of 524 patients with COVID-19 in Henan Province. J. Tradit. Chin. Med. 61, 1391–1396. 10.1007/s11684-020-0803-8

[B27] Li Q. Bai C. Weiying X. Pang X. Liu S. Liu T. (Forthcoming 2020c). Deciphering the pharmacological mechanisms of ma xing shi Gan decoction against COVID-19 through integrating network pharmacology and experimental validation. Front. Pharmacol. 10.3389/fphar.2020.581691 PMC7725906 33324213

[B28] Li Y. Jerkic M. Slutsky A. S. Zhang H. (2020). Molecular mechanisms of sex bias differences in COVID-19 mortality. Crit. Care. 24, 405. 10.1186/s13054-020-03118-8 32646459 PMC7347256

[B29] Li J. (2020a). Safety and efficacy of the Maxing Shigan decoction plus moxifloxacin injection on community acquired pneumonia. Clinical J. Chin. Med. 12, 52–54. 10.3969/j.issn.1674-7860.2020.14.017

[B30] Lin S.-Y. X. L. Z. Y.-Z. (2015). Influence of modified Maxing Shigan Decoction on TGF-β/Smad pathway in acute radiation lung injury. China J. Trad. Chin. Med. Pharm. 30, 4117–4119.

[B31] Lin Y. C. Chang C. W. Wu C. R. (2016). Antitussive, anti-pyretic and toxicological evaluation of Ma-Xing-Gan-Shi-Tang in rodents. BMC Compl. Alternative Med. 16, 456. 10.1186/s12906-016-1440-2 PMC5105301 27832784

[B32] Liu Z. Liang S. (2014). Preliminary study on anti-inflammatory effect of Phragmites decoction on mice. Guide of China Medicine. 12, 61–62. 10.15912/j.cnki.gocm.2014.34.039

[B33] Luo H. Tang Q.-L. Shang Y.-X. Liang S.-B. Yang M. Robinson N. (2020a). Can Chinese medicine be used for prevention of corona virus disease 2019 (COVID-19)? A review of historical classics, research evidence and current prevention programs. Chin. J. Integr. Med. 26, 243–250. 10.1007/s11655-020-3192-6 32065348 PMC7088641

[B34] Luo H. Yang M. Tang Q. L. Hu X. Y. Willcox M. L. Liu J. P. (2020b). Characteristics of registered clinical trials on traditional Chinese medicine for coronavirus disease 2019 (COVID-19): a scoping review. Eur J Integr Med, 41, 101251. 10.1016/j.eujim.2020.101251 33204368 PMC7659925

[B35] Ma C. Cheng P. Li X. (2020). Research progress on chemical composition and pharmacological activities of patchouli. J. Chengdu Univ. TCM. 43, 72–80. 10.13593/j.cnki.51-1501/r.2020.01.072

[B36] Magro G. (2020). Cytokine Storm: is it the only major death factor in COVID-19 patients? Coagulation role. Med. Hypotheses. 142, 109829. 10.1016/j.mehy.2020.109829 32428809 PMC7217113

[B37] Miao S. M. Zhang Q. Bi X. B. Cui J. L. Wang M. L. (2020). A review of the phytochemistry and pharmacological activities of Ephedra herb. Chin. J. Nat. Med. 18, 321–344. 10.1016/S1875-5364(20)30040-6 32451091

[B38] Pan B. Fang S. Zhang J. Pan Y. Liu H. Wang Y. (2020). Chinese herbal compounds against SARS-CoV-2: puerarin and quercetin impair the binding of viral S-protein to ACE2 receptor. Comput. Struct. Biotechnol. J. 18, 3518–3527. 10.1016/j.csbj.2020.11.010 33200026 PMC7657012

[B39] Panigada M. Bottino N. Tagliabue P. Grasselli G. Novembrino C. Chantarangkul V. (2020). Hypercoagulability of COVID-19 patients in intensive care unit: a report of thromboelastography findings and other parameters of hemostasis. J. Thromb. Haemostasis. 18, 1738–1742. 10.1111/jth.14850 32302438 PMC9906150

[B40] Perwaiz S. a. J. S. (2014). “Aristolochic acids,” in Encyclopedia of toxicology. Editor Wexler P. (Ottawa: Marketed Health Products Directorate), 298–301.

[B41] Petrosillo N. Viceconte G. Ergonul O. Ippolito G. Petersen E. (2020). COVID-19, SARS and MERS: are they closely related?. Clin. Microbiol. Infect. 26, 729–734. 10.1016/j.cmi.2020.03.026 32234451 PMC7176926

[B42] Ragab D. Salah Eldin H. Taeimah M. Khattab R. Salem R. (2020). The COVID-19 cytokine storm; what we know so far. Front. Immunol. 11, 1446. 10.3389/fimmu.2020.01446 32612617 PMC7308649

[B43] Ramalingam M. Kim H. Lee Y. Lee Y. I. (2018). Phytochemical and pharmacological role of liquiritigenin and isoliquiritigenin from radix glycyrrhizae in human health and disease models. Front. Aging Neurosci. 10, 348. 10.3389/fnagi.2018.00348 30443212 PMC6221911

[B44] Roback J. D. Guarner J. (2020). Convalescent plasma to treat COVID-19: possibilities and challenges. J. Am. Med. Assoc. 323, 1561–1562. 10.1001/jama.2020.4940 32219429

[B45] Ruan Q. Yang K. Wang W. Jiang L. Song J. (2020). Clinical predictors of mortality due to COVID-19 based on an analysis of data of 150 patients from Wuhan, China. Intensive Care Med. 46, 846–848. 10.1007/s00134-020-05991-x 32125452 PMC7080116

[B46] Ruben M. D. Smith D. F. Fitzgerald G. A. Hogenesch J. B. (2019). Dosing time matters. Science. 365, 547–549. 10.1126/science.aax7621 31395773 PMC8011856

[B47] Ruscitti P. Berardicurti O. Di Benedetto P. Cipriani P. Iagnocco A. Shoenfeld Y. (2020). Severe COVID-19, another piece in the puzzle of the hyperferritinemic syndrome. An immunomodulatory perspective to alleviate the storm. Front. Immunol. 11, 1130. 10.3389/fimmu.2020.01130 32574264 PMC7270352

[B48] Scully E. P. Haverfield J. Ursin R. L. Tannenbaum C. Klein S. L. (2020). Considering how biological sex impacts immune responses and COVID-19 outcomes. Nat. Rev. Immunol. 20, 442–447. 10.1038/s41577-020-0348-8 32528136 PMC7288618

[B49] Shi D. Liu D. (2018). Research progress on pharmacological effects and processing technology of Armeniacae Semen Amarum. Asia-Pacific Tradit. Med. 14, 106–109. 10.11954/ytctyy.201812036

[B50] Siddiqi H. K. Mehra M. R. (2020). COVID-19 illness in native and immunosuppressed states: a clinical-therapeutic staging proposal. J. Heart Lung Transplant. 39, 405–407. 10.1016/j.healun.2020.03.012 32362390 PMC7118652

[B51] Song W. Ni S. Fu Y. Wang Y. (2018). Uncovering the mechanism of Maxing Ganshi Decoction on asthma from a systematic perspective: a network pharmacology study. Sci. Rep. 8, 17362. 10.1038/s41598-018-35791-9 30478434 PMC6255815

[B52] Song X. Ren T. Zheng Z. Lu T. Wang Z. Du F. (2017). Anti-tumor and immunomodulatory activities induced by an alkali-extracted polysaccharide BCAP-1 from Bupleurum chinense via NF-κB signaling pathway. Int. J. Biol. Macromol. 95, 357–362. 10.1016/j.ijbiomac.2016.10.112 27884671

[B53] Spiezia L. Boscolo A. Poletto F. Cerruti L. Tiberio I. Campello E. (2020). COVID-19-Related severe hypercoagulability in patients admitted to intensive care unit for acute respiratory failure. Thromb. Haemostasis. 120, 998–1000. 10.1055/s-0040-1710018 32316063 PMC7295272

[B54] Spinner C. D. Gottlieb R. L. Criner G. J. Arribas López J. R. Cattelan A. M. Soriano Viladomiu A. (2020). Effect of Remdesivir vs standard care on clinical status at 11 Days in patients with moderate COVID-19: a randomized clinical trial. J. Am. Med. Assoc. 324, 1048–1057. 10.1001/jama.2020.16349 PMC7442954 32821939

[B55] Tang N. Bai H. Chen X. Gong J. Li D. Sun Z. (2020a). Anticoagulant treatment is associated with decreased mortality in severe coronavirus disease 2019 patients with coagulopathy. J. Thromb. Haemostasis. 18, 1094–1099. 10.1111/jth.14817 32220112 PMC9906401

[B56] Tang N. Li D. Wang X. Sun Z. (2020b). Abnormal coagulation parameters are associated with poor prognosis in patients with novel coronavirus pneumonia. J. Thromb. Haemostasis. 18, 844–847. 10.1111/jth.14768 32073213 PMC7166509

[B57] Tao Q. Du J. Li X. Zeng J. Tan B. Xu J. (2020). Network pharmacology and molecular docking analysis on molecular targets and mechanisms of Huashi Baidu formula in the treatment of COVID-19. Drug Dev. Ind. Pharm. 46, 1345–1353. 10.1080/03639045.2020.1788070 32643448 PMC7441778

[B58] Tsou L. K. Lara-Tejero M. Rosefigura J. Zhang Z. J. Wang Y. C. Yount J. S. (2016). Antibacterial flavonoids from medicinal plants Covalently inactivate type III protein secretion substrates. J. Am. Chem. Soc. 138, 2209–2218. 10.1021/jacs.5b11575 26847396 PMC4831573

[B59] Wang B. Wang L. Kong X. Geng J. Xiao D. Ma C. (2020a). Long-term coexistence of SARS-CoV-2 with antibody response in COVID-19 patients. J. Med. Virol. 92, 1684–1689. 10.1002/jmv.25946 32343415 PMC7267623

[B60] Wang C. Xie J. Zhao L. Fei X. Zhang H. Tan Y. (2020b). Alveolar macrophage dysfunction and cytokine storm in the pathogenesis of two severe COVID-19 patients. EBioMedicine. 57, 102833. 10.1016/j.ebiom.2020.102833 32574956 PMC7305897

[B61] Wang Y. Zhang D. Du G. Du R. Zhao J. Jin Y. (2020c). Remdesivir in adults with severe COVID-19: a randomised, double-blind, placebo-controlled, multicentre trial. Lancet. 395, 1569–1578. 10.1016/S0140-6736(20)31022-9 32423584 PMC7190303

[B62] Wang C. W.S. Jiang L. Xu W. Yang Y. Hu J. (2020). Comprehensive analysis of TCM diagnosis and treatment Schemes for COVID-19 in all regions of China. Modernization of Tradit. Chin. Med. and Materia Medica-World Science and Technology. 22, 257–263. 10.1016/S0140-6736(20)31022-9

[B63] Wei P. Ma Q. Ren M. Luo J. (2016a). Influence of compatibility on tissue distribution of ephedra alkaloids of the drug pairsof Herba Ephedrae and Radix Glycyrrhizae in rats. J. Pharmaceut. Res. 35, 187–192. 10.13506/j.cnki.jpr.2016.04.001

[B64] Wei P. Zheng F. Huo H. Chen F. Luo J. (2016b). Effect of different compatibility proportion of drug pair of ephedrae herba-glycyrrhizae radix et rhizoma on plasma pharmacokinetics of alkaloids from ephedrae herba. Chin. J. Experimental Tradit. Med. Formulae. 22, 100–105. 10.13422/j.cnki.syfjx.2016070100

[B65] Worley C. Lindbloom E. (2003). Ephedra and ephedrine: modest short-term weight loss, at a price. J. Fam. Pract. 52, 518–520. 12841963

[B81] Xin S. Cheng X. Zhu B. Liao X. Yang F. Song L. (2020). Clinical retrospective study on the efficacy of Qingfei Paidu decoction combined with Western medicine for COVID-19 treatment. Biomed. Pharmacother. 129, 110500. 32768975 10.1016/j.biopha.2020.110500 PMC7342031

[B82] Xiong W. Z. Wang G. Du J. Ai W. (2020). Efficacy of herbal medicine (Xuanfei Baidu decoction) combined with conventional drug in treating COVID-19:a pilot randomized clinical trial. Integr. Med. Res. 9, 100489. 32874913 10.1016/j.imr.2020.100489 PMC7452296

[B66] Yang L. Z. Chunyang, Cai J. Chen S. Jiang M. (2019). Preventive effect of tonic Chinese medicine on radiation pneumonia: a systematic reviews. Herald of Medicine. 38, 1069–1078. 10.3870/j.issn.1004-0781.2019.08.019

[B68] Yao J. S. X. Chen Q. Fan S. Yang R. Peng B. (2020). Theoretical study on corona virus disease 2019 treated by qingfei paidu decoction. Liaoning J. Tradit. Chin. Med. 47, 94–98. 10.13192/j.issn.1000-1719.2020.05.029

[B69] Yin X. (2019). Study on the anti-inflammatory mechanism of gypsum based on Ca and other inorganic elements. PhD thesis. Beijing (China): Beijing University of Chinese Medicine.

[B70] Yu S. Bai M. Miao M. (2013). Modern research of blood-activating traditional Chinese medicine. Acta Chinese Medicine. 28, 1692–1694. 10.16368/j.issn.1674-8999.2013.11.061

[B71] Zakaryan H. Arabyan E. Oo A. Zandi K. (2017). Flavonoids: promising natural compounds against viral infections. Arch. Virol. 162, 2539–2551. 10.1007/s00705-017-3417-y 28547385 PMC7087220

[B72] Zhang B. Ling L. Lu F. Dai B. Tan L. Xiao Z. (2013). Lung inflammation and neuraminidase activity of Maxing Shigan Decoction on mice which were infected by influenza virus. Chin. J. Tradit. Chin. Med. and Pharmacy. 28, 1094–1099.

[B73] Zhang D. H. Wu K. L. Zhang X. Deng S. Q. Peng B. (2020). In silico screening of Chinese herbal medicines with the potential to directly inhibit 2019 novel coronavirus. J Integr Med. 18, 152–158. 10.1016/j.joim.2020.02.005 32113846 PMC7102521

[B80] Zhang Y. Yang H. He X. (2020). The Curative mechanism of maxing shigan decoction on cytokine storm of COVID19 based on network pharmacology. World Chinese Med. 15, 1908–1913. 10.3969/j.issn.1673-7202.2020.13.011

[B74] Zhang Y. Z. Holmes E. C. (2020). A genomic perspective on the origin and emergence of SARS-CoV-2. Cell. 181, 223–227. 10.1016/j.cell.2020.03.035 32220310 PMC7194821

[B75] Zhao Z. Li Y. Zhou L. Zhou X. Xie B. Zhang W. (2020). Prevention and treatment of COVID-19 using Traditional Chinese Medicine: A review. Phytomedicine, 153308. 10.1016/j.phymed.2020.153308 32843234 PMC7439087

[B76] Zhong C. Wang Y. Li X. Ren L. Zhao J. Hu Y. (2020). Clinical features of patients infected with 2019 novel coronavirus in Wuhan, China. Lancet 395, 497–506. 10.1016/S0140-6736(20)30183-5 31986264 PMC7159299

[B77] Zhou X. Xuexin L. Xiong Z. (2020). Modified maxing shigan decoction for prevention and treatment of 29 cases of radiation pneumonitis caused by synchronous radiotherapy and chemotherapy. Zhejiang J. Tradit. Chin. Medic. 55, 730–731. 10.3389/fphar.2020.560448

[B78] Zhou Z. Gao N. Wang Y. Chang P. Tong Y. Fu S. (2020). Clinical studies on the treatment of novel coronavirus pneumonia with traditional Chinese medicine—a literature analysis. Front. Pharmacol. 11, 1355. 10.3389/fphar.2020.560448 PMC7511712 33013397

